# Implementing point-of-care tests to optimize antibiotic use for vaginal discharge: a study protocol for a randomized controlled trial in Nepal

**DOI:** 10.1186/s13063-025-09333-4

**Published:** 2025-12-12

**Authors:** S. Shrestha, S. Shakya, J. J. Infanti, E. Skovlund, M. R. Simpson, R. A. M. Lonnee-Hoffmann

**Affiliations:** 1 Department of Clinical and Molecular Medicine, Faculty of Medicine and Health Sciences, Norwegian University of Science and Technology (NTNU), Trondheim, Norway; 2 Department of Obstetrics and Gynecology, Dhulikhel Hospital/Kathmandu University Hospital, Dhulikhel, Nepal; 3 Department of Obstetrics and Gynecology, Kathmandu University School of Medical Sciences, Dhulikhel, Nepal; 4 Department of Public Health and Nursing, Faculty of Medicine and Health Sciences, Norwegian University of Science and Technology (NTNU), Trondheim, Norway; 5 Department of Obstetrics and Gynecology, St. Olavs University Hospital, Trondheim, Norway

**Keywords:** Point-of-care tests, Vaginal discharge, Antimicrobial resistance, Randomized controlled trial, Syndromic management, Sexually transmitted infections, Nepal, Domestic violence, Anxiety and depression

## Abstract

**Background:**

Vaginal discharge (VD) is a common health concern among women of reproductive age worldwide. In low- and lower-middle-income countries (LLMICs), one in four women experiences distressing VD which may indicate underlying infections. About one-third of these cases are caused by one of three curable sexually transmitted infections (STIs)*—Chlamydia trachomatis [Ct]*, *Neisseria gonorrhea [Ng], Trichomonas vaginalis [Tv]*—or by bacterial vaginosis (BV). Syndromic management, the standard approach to care in LLMICs, relies on empirical antibiotic treatment, often resulting in overtreatment and contributing to antimicrobial resistance. To reduce unnecessary use of antibiotics, affordable, accurate, and rapid diagnostic tests such as point-of-care tests (POCTs) are needed—particularly in settings where over-the-counter antibiotic use is common and psychosocial problems may be somatized. Additionally, education on VD and appropriate antibiotic use, along with recognition of mental health or domestic violence issues, may be important for facilitating acceptance of POCTs.

**Methods:**

This study is a randomized controlled double-blind, superiority trial with follow-up assessments at 4 weeks and 4 months conducted in a Nepalese teaching hospital and its outreach centers. Participants are randomly assigned to one of three study arms: Arm 1 receives treatment, based on syndromic management; Arm 2 receives POCT-guided treatment using the *Cepheid GeneXpert®* (for *Ct* and *Ng*), pH and whiff tests (for BV and *Tv*), and the *OSOM®* test (for *Tv*); Arm 3 receives the same POCT-guided treatment as Arm 2, plus an educational intervention, and referral for psychosocial concerns. The primary outcome is the proportion of participants overtreated with antibiotics at the primary consultation, comparing Arm 1 versus Arms 2 and 3. Secondary outcomes include over-the-counter antibiotic acquisition, subsequent health-seeking behavior, and changes in VD symptom development over time.

**Discussion:**

The trial assesses the effectiveness and impact of integrating POCTs into VD management in a resource-limited setting. Results of comparing syndromic-based management with POCT-guided diagnostic testing and treatment, both alone and combined with psychosocial and education-based interventions, inform strategies to reduce antibiotic overuse and improve broader reproductive health concerns.

**Trial registration:**

ClinicalTrials.gov NCT05977491. Registered on 8 April 2023. https://clinicaltrials.gov/study/NCT05977491?cond=vaginal%20discharge&rank=6

**Protocol version:**

Version 6, October 30, 2025.

**Supplementary Information:**

The online version contains supplementary material available at 10.1186/s13063-025-09333-4.

## Background

Women of reproductive age are frequently bothered by vaginal discharge (VD), leading many to seek care at gynecological outpatient departments (OPDs) [1]. VD can be physiological or pathological. Hormonal fluctuations influence physiological VD, while sexually transmitted infections (STIs), candidiasis, and bacterial vaginosis (BV) are the most common pathological causes of abnormal VD. All these conditions typically involve changes in the color, consistency, amount, or odor of the discharge [2].

Approximately one-quarter of adult women in South Asia report bothersome VD for which they seek medical care [3, 4]. A population-based study in Nepal found that while 34% of women self-reported bothersome VD, only 25.7% exhibited abnormal physical findings on gynecological examination [5]. In a tertiary hospital OPD setting in Nepal, an unpublished study found self-reported VD concerns in 28% of women, comparable to the 24.6% prevalence reported in a similar setting in India [6, 7].

Among women presenting with bothersome VD in clinical settings, approximately one-third are found to have one or more of three treatable STIs—*Chlamydia Trachomatis* (*Ct*), *Neisseria gonorrhea* (*Ng*), *Trichomonas vaginalis (Tv)*—or to have BV, which is caused by an imbalance of the vaginal flora and is not currently classified as an STI [8–10]. STIs and their aftereffects are common in low- and lower-middle-income countries (LLMICs), particularly in Southeast Asia. In a population-based study conducted in Nepal, curable STI pathogens were detected in 6.8 to 9.4% of women who either reported abnormal VD or had it confirmed by gynecological examination [5, 11–13].

In 1984, the World Health Organization (WHO) endorsed syndromic management guidelines for STIs in LLMICs, where etiologic STI diagnosis is often unavailable. This comprehensive and cost-effective approach relies on observed signs, symptoms, and risk factors to guide empirical treatment for *Ct*, *Ng*, *Tv,* BV, and candidiasis at the initial point of contact with the health care system [14, 15]. To date, syndromic management remains the standard of care for STIs in Nepal [15]. However, this approach has particularly low diagnostic accuracy for cervical infections caused by *Ct* and *Ng*, often leading to overtreatment with broad-spectrum antibiotics such as cephalosporins, azithromycin, and doxycycline. This has raised concerns about antimicrobial resistance (AMR), especially in *Ng*, where treatment options are limited [14]. AMR has been identified by the WHO as one of the top global health threats [16], and *Ng* is now designated a high-priority AMR pathogen in the 2024 WHO bacterial priority list [17]. These limitations underscore the need for accurate diagnostic tools and more targeted antibiotic use.

To address these challenges, the WHO’s 2021 *Global Health Sector Strategy* recommends etiological STI treatment wherever feasible, as part of the goal to end the STI epidemic by 2030 [18, 19]. While molecular tests are considered the gold standard for STI diagnosis, their use has been largely limited to high-income countries due to high cost, infrastructure requirements (such as the need for laboratory facilities), need for trained personnel, and lengthier turnaround time for results [18, 20]. The WHO has therefore advocated for point-of-care tests (POCTs) that preferably fulfill the ASSURED criteria—affordable, sensitive, specific, user-friendly, rapid and robust, equipment-free, and deliverable to end users [21]. POCTs offer fast results and allow more precise antibiotic treatment, potentially benefiting both patients and sexual partners. Highly accurate POCTs based on nucleic acid amplification tests (NAATs) offer 90–100% sensitivities and specificities, though they remain quite expensive [22]. The Cepheid *Ct/Ng* molecular POCT, for example, has demonstrated over 95% sensitivity and specificity, with a 90-minute turnaround time [22]. However, cost remains a barrier, particularly in LLMICs. To address affordability while maintaining diagnostic accuracy, alternative types or combinations of POCTs may be needed [14]. Extremely low-cost, non-molecular POCTs such as pH testing and confirmatory whiff tests have shown sensitivity and specificity above 87% for BV. However, sensitivity for *Tv* remains low (51.8%), though specificity is higher (78.8%) [23, 24]. Antibody-based POCTs such as the *OSOM®* test offer better sensitivity and specificity and remain relatively affordable for *Tv* detection [25].

Despite these advantages, POCTs have not been widely implemented. Barriers include affordability, lack of quality assurance systems, insufficient clinician support, site-specific logistical issues, and limited awareness among clinicians and patients [26–28]. Over-the-counter (OTC) antibiotic access also complicates efforts to control AMR; in many settings, more than half of antibiotics are purchased from a pharmacy without a medical prescription, contributing to the growing burden of AMR [29, 30]. Even if POCTs reduce unnecessary prescriptions of antibiotics, it is unclear whether women will adhere to restrictive treatment advice in South Asia, where reproductive health literacy is often low [31]. No studies to date have examined whether education on VD and antibiotic use might enhance treatment adherence or POCT acceptability. Moreover, a large proportion of women reporting bothersome VD do not have STIs [5]. In such cases, symptoms may reflect underlying common mental health conditions or trauma from experiences of sexual or domestic violence (DV) [3, 32–34]. Addressing these factors could improve VD management overall and help reduce unnecessary antibiotic use.

## Methods/design

### Aim

This study evaluates whether implementing a combination of highly accurate and simple POCTs for the management of bothersome VD in healthcare settings in Nepal can reduce antibiotic overtreatment compared to the syndromic management approach. It also examines the impact of incorporating education and attention to psychosocial concerns on outcomes such as OTC purchase of antibiotics, symptom development, and patient satisfaction. In addition, the study assesses the diagnostic accuracy of a combination of POCTs for *Tv* and BV—both combined and individually.

### Trial design

**Fig. 1:**
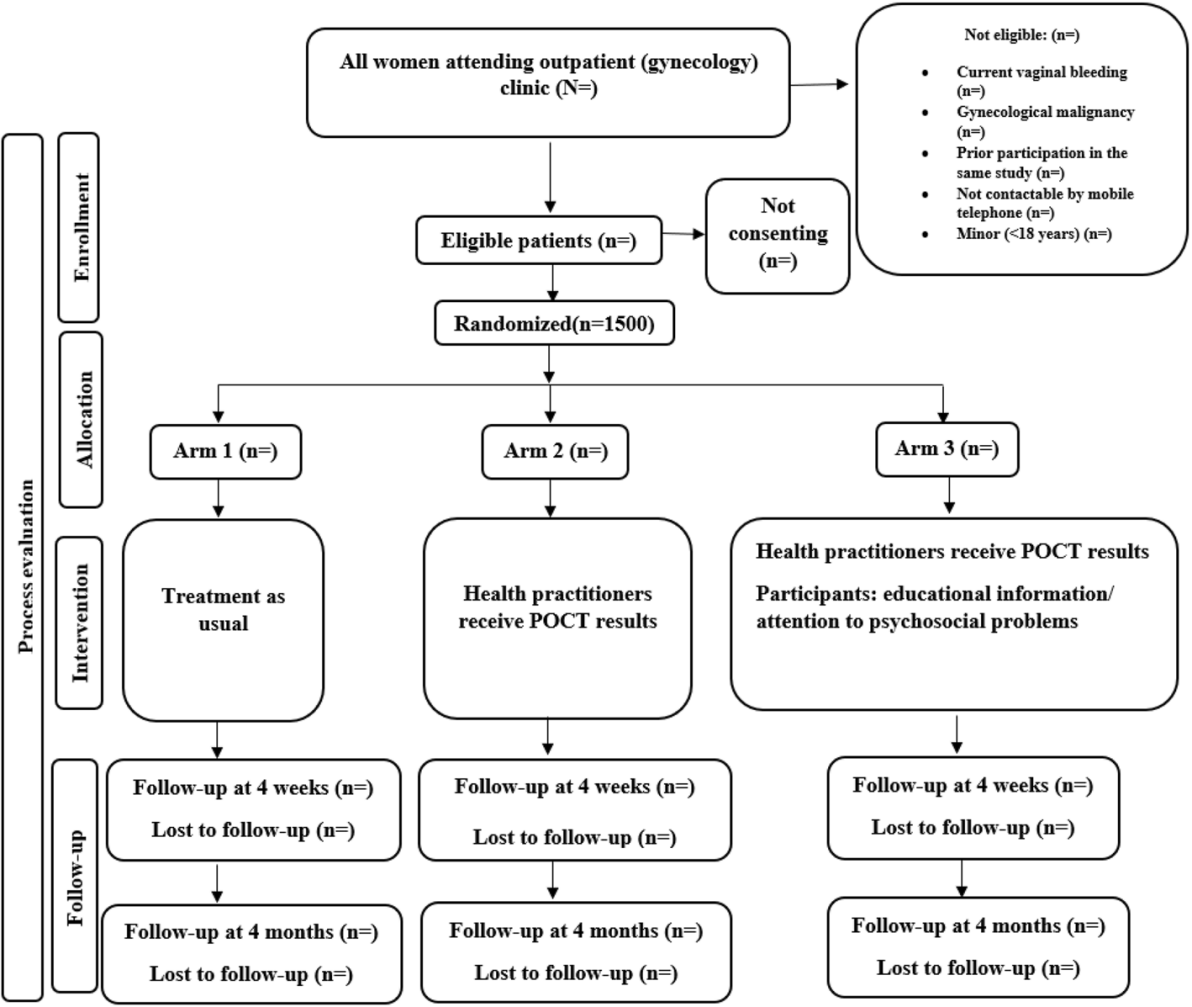
Consort diagram: Flow of participants from recruitment to follow-up

This study is a randomized, controlled, double-blinded superiority trial. Study participants are randomly allocated into three parallel arms in a 1:1:1 ratio. The trial arms are as follows: 1. Treatment as usual, 2. Treatment based on POCT results, and 3. Treatment based on POCT results combined with education about VD and antibiotics and addressing psychosocial vulnerabilities (see Fig. 1).

### Study setting

The study is conducted at Dhulikhel Hospital (DH) and seven of its outreach centers (ORCs) in Bagmati province, Nepal. DH is a non-profit, independent, non-governmental, tertiary hospital located in a semi-urban area of Nepal. It provides healthcare services to approximately 2.7 million people from diverse ethnic backgrounds across the country through 18 ORCs at the grassroots level [35]. The seven ORCs involved in the study were selected based on facility observation checklists (distance from DH, staffing, patient volume, infrastructure) with the aim of being representative of rural Nepal.

At DH, women presenting with concerns about VD are seen by obstetrics and gynecology consultants and resident doctors at the OPD. At the ORCs, these patients are attended by medical officers or obstetrics and gynecology residents.

### Participants

#### Eligibility criteria

Women 18 years and above who present with a main concern about VD at gynecology OPDs at DH or one of the selected ORCs. Exclusion criteria include current vaginal bleeding, known or suspected gynecological malignancy, prior participation in the same study, refusal to give informed consent, and unavailability of mobile phone contact for follow-up.

#### Recruitment and consent processes

The research assistant (RA) informs all women attending the gynecology OPD before 13:00 briefly about the study and checks for eligibility. Women expressing interest receive a detailed explanation, and written informed consent is obtained via signature or fingerprint with a witness for participants with limited literacy. The use of participant data and biological specimens is explicitly described.

### Interventions

#### Overview and rationale

The intervention targets the most clinically significant pathogens associated with abnormal VD: *Ct, Ng, Tv*, and BV. Untreated *Ct* and *Ng* can lead to serious health consequences, while overtreatment is associated with growing AMR. Although *Tv* and BV typically cause less severe complications, they impose a high symptom burden and are common among women presenting with bothersome VD. Both are treated with the same narrow-spectrum antibiotic, which is less likely to cause AMR [14, 36].

The intervention combines a highly accurate molecular test for *Ct* and *Ng* and a simpler POCT for *Tv* and BV, aiming to optimize diagnostic performance and resource use in Nepalese healthcare settings.

#### Trial arms

The trial consists of three parallel arms:

- Arm 1: Standard care (syndromic management). Health practitioners prescribe treatment based on clinical symptoms and signs, following Nepal’s national guidelines [15]. POCT results are not made available.
- Arm 2: POCT-guided treatment. Health practitioners receive POCT results for *Ct, Ng, Tv*, and BV and use this information to guide decisions in prescribing antibiotics.
- Participants under Arm 2 and Arm 1 view a general health education video on hand hygiene and dengue fever, produced by Save the Children, the Ministry of Health and Population, and Nepal Television.
- Arm 3: POCT-guided treatment plus psychosocial intervention. In addition to the POCT-guided treatment (as in Arm 2), participants in Arm 3 receive: an educational intervention consisting of a short video and leaflet with basic information on VD and appropriate antibiotic use. This intervention was developed using qualitative input from user groups, expert consultations, and with a local artist (article forthcoming). The participants are also screened for symptoms of anxiety and depression using content- and language-validated questionnaires. Those who screen positive are offered psychological counseling. In addition, all participants in this arm are asked whether they would like to be referred to a crisis management center for support related to DV [37–39].

#### POCT methods

a For *Ct* and *Ng*, self-collected urine samples are analyzed using the *Cepheid GeneXpert®* molecular test, administered by RAs. Results are reported as positive or negative for *Ct* and/or *Ng* and shared with the attending health practitioner. The test is validated and demonstrates high sensitivity and specificity [22].
b For the initial diagnostic approach to *Tv* and BV, each participant self-collects a vaginal discharge sample using a swab. The RA then measures the pH of the sample; if the pH is above 4.5, a drop of potassium hydroxide (KOH) is added to perform the whiff test, where a fishy odor indicates a positive result [23]. The health practitioner is subsequently informed of the pH level (above or below 4.5) and whether the whiff test result is positive or negative.
c For the diagnostic approach after protocol adaptation (see 2.5.4); for VD with pH > 4.5 and a negative whiff test, an antigen-based Tv test (*OSOM®*) is applied. Results from this test (positive/negative) are shared with the health practitioner.

#### Protocol adaptation for Tv testing

An unplanned interim analysis was conducted after 218 participants had been enrolled, prompted by concerns that many laboratory-confirmed *Tv* cases had not received treatment due to negative POCT results. Without unblinding or compromising randomization, only the POCT results and gold standard laboratory results for *Tv* were compared. This analysis showed low sensitivity of the pH and whiff test: initial POCT approach (3.7% correctly identified; 7.8% false negatives). For ethical reasons, affected participants were traced and treated, and an additional diagnostic step was added for the remainder of the trial (see “ POCT methods” section).

Although *OSOM®* is a validated diagnostic tool for *Tv* detection, its sequential use following pH and whiff testing has not previously been evaluated [25].

#### Comparisons

The trial compares the accuracy of antibiotic prescriptions between the current /syndromic and POCT-guided treatment arms. It also evaluates whether the addition of educational and psychosocial support improves adherence to treatment recommendations, influences health-seeking behavior, and affects symptom resolution and patient satisfaction.

#### Implementation and adherence strategies

Before the trial began, participating health practitioners received brief training on study procedures, POCT accuracy, and updates to Nepal’s national syndromic management guidelines for STIs. This training is repeated twice during the trial.

To ensure ethical standards of care, participants in all arms who test positive for an STI via gold-standard laboratory tests are offered appropriate treatment, even if this was not prescribed initially.

As POCT procedures may prolong clinic visits by up to two hours, participants are offered refreshments during the waiting period.

HPs are free to provide any concomitant care as they find indicated and shall assess the participants independently of the study.

### Outcomes

The primary outcome measures are:

- The proportion of participants overtreated with antibiotics at the primary consultation with the health practitioner Overtreatment is defined as receiving a cephalosporin and/or macrolide when *Ng* is negative; a tetracycline or macrolide when *Ct* is negative; or a nitroimidazole when the gold standard test for *Tv* or BV is negative, (medications as specified in the Nepali STI case management guidelines) [15] (comparing arm 1 with the combined arms 2 and 3).
- The proportion of participants prescribed AMR driving antibiotics (Cephalosporins, Azithromycin, Ciprofloxacin) at the primary consultation with the health practitioner. comparing treatment as usual (arm 1) with POCT-based treatment (arms 2 and 3).
- The proportion of participants adhering to treatment recommendations, comparing arms 2 and 3. Adherence is assessed during telephone follow-ups after one month and is defined as follows: (a) participants report that they took the prescribed medication; (b) no additional purchase of antibiotics; and (c) no purchase of other medication for VD.

The study also examines six secondary outcome measures at various time points. The baseline data from this trial will also contribute to the analysis of the epidemiology of VD and infection including psychosocial factors, and measure the diagnostic accuracy of the POCT.

### Sample size

Based on the study objectives, recruitment feasibility, and the desired precision of estimates (i.e., two-sided 95% confidence intervals [CIs]), the sample size was calculated using PASS Sample Size software. Antibiotic use will be compared between both Arm 2 and Arm 3 versus Arm 1 (*N* = 1500) at the first consultation and will serve as a secondary outcome. The effect of the educational measures on the use of OTC antibiotics will be compared between Arm 2 and Arm 3 (*n* = 1000) at 4 weeks and 4 months of follow-up.

It is estimated that approximately 85% of women in LLMICs are over-treated with antibiotics for VD, and around 50% receive antibiotics that contribute to AMR. Furthermore, about 40% of women are expected to purchase antibiotics OTC. The overtreatment with antibiotics and the use of AMR will be assessed at the first consultation whilst the adherence to the treatment recommendation will be assessed at 4 weeks as primary outcome and at 4 weeks and 4 months of follow-up as secondary outcome.

With 1500 participants randomized equally across the three arms (allocation ratio of 1:1:1), a 10–20 percentage point difference between groups in any of these measures will produce CIs with a width of 8–12%, achieving at least 90% power for each comparison at a significance level of 0.017 (adjusted from 0.05 to account for multiplicity with three primary outcome measures). For the assessment of over-the-counter antibiotic use, even with a 40% loss to follow-up, the expected CI width remains around 15%.

### Randomization and blinding

After obtaining informed consent, RAs open consecutively numbered, sealed opaque envelopes containing the randomized group assignment. Randomization is computer-generated using a simple randomization method (Research Randomizer©). Participants are blinded to their group assignment. Each PID (1–1500) was assigned to one of the three trial arms (1, 2, 3) based on pseudo-random numbers uniformly distributed between 0 and 1, with equal probability of allocation to each group.

The resulting allocation list was generated by an independent researcher and implemented on site using consecutively numbered, sealed opaque envelopes containing the randomized group assignment. We opted not to stratify or block to keep field procedures straight forward and robust in this multi-site and resource-limited setting. This reduced the risk of errors in envelope handling and simplified implementation for rotating staffs. Due to the nature of the intervention, only RAs performing follow-up and the statistician are blinded to group allocation. HPs are informed about whether they will receive POCT results but blinded as to whether participants receive educational materials or are offered counseling (arm 1 vs. arm 2 and 3).

### Data collection

#### Assessment and data collection procedures

The study’s RAs receive proficiency training before data collection, including a one-day course on Good Clinical Practice. After randomization, participants are provided with verbal and pictorial instructions to guide the self-collection of vaginal swabs and urine samples. Following sample collection, participants complete a study questionnaire using an electronic tablet equipped with a headset, utilizing a Color-coded Audio Computer-Assisted Self Interview (C-ACASI) system [40]. After completing the questionnaire, participants proceed to consultation with a health practitioner.

#### Prescriptions and antibiotic use

The health practitioner completes a questionnaire documenting the antibiotics prescribed—either after the consultation (arm 1) or upon receiving POCT results (arms 2 and 3). If the health practitioner delays prescription while awaiting other tests (e.g., urine), the RA follows up to ensure completion of the questionnaire. These data are entered daily into the case report form (CRF). If insufficient treatment is provided based on gold-standard STI results, this is recorded after the initial prescription is documented, and any additional antibiotics provided are also registered.

#### Laboratory diagnostics

Gold standard STI testing includes the following: *Cepheid GeneXpert*® for *Ct*, *Ng,* and *Tv*. For BV, dried wet mounts from vaginal smears are collected for Nugent scoring and wet mount microscopy. All tests are performed by RAs, except Nugent scoring (performed by microbiologist SRM) and wet mount microscopy (performed by RLH). Nugent scoring is considered the gold standard for BV. For quality control, 10% of slides are double read by a second microbiologist (JEA).

#### Baseline data and secondary outcomes

Baseline data are collected using a C-ACASI with RAs available to assist. This ensures the inclusion of participants with limited literacy. Data are uploaded to a secure cloud and later transferred to the CRF. These data are acquired as follows:

Questions adapted from: 

- WHO clinical study guidance on POCT for VD to establish sociodemographic profile, reproductive and sexual history, and the VD symptoms [41].
- The Hopkin’s Symptom Checklist for screening anxiety and depression [37, 38].
- DV screening questions adapted from the 2016 Nepal Demographic Health Survey, which have been validated in Nepali [39].
- Health practitioners’ documentation of clinical findings on paper questionnaires, which are collected and transferred to the CRF [41].

Secondary outcome data are collected during telephone follow-ups at one- and four-month post-consultation. These follow-ups are conducted using printed questionnaires by RAs who are blinded to group assignments. The information is later entered into the CRF by a researcher.

#### Retention and follow-up

Participants are contacted via telephone up to four times at each follow-up point to maximize retention and reduce loss to follow-up.

#### Process evaluation

A process evaluation is conducted at multiple time points throughout the study’s inclusion period and involves in-depth interviews with participants at DH and the ORCs, focus group discussions with the health practitioners from the obstetrics and gynecology department (pre- and post-trial), stakeholder focus groups, and interviews with the RAs at the end of the RCT.

#### Participant timeline

**Fig. 2:**
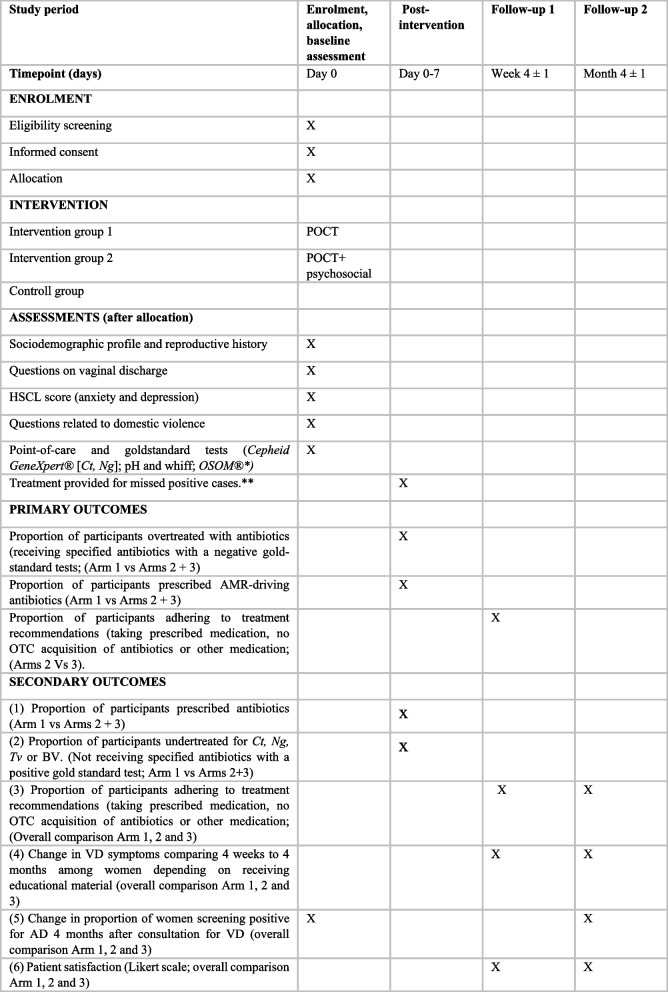
Participant timeline for enrolment, intervention, and assessment. *Gold standard test for bacterial vaginosis is Nugent scoring, which is performed within 6 months on air-dried microscopy slides. **Treatment for missed positive cases (*Ct, Ng, Tv)* is provided after collection of main outcomes (prescription of antibiotics by health practitioners). Abbreviations: AD, anxiety and/or depression; AMR, antimicrobial resistance; Ct, *Chlamydia trachomatis*; DV, domestic violence; HSCL, Hopkins Symptom Checklist; Ng, *Neisseria gonorrhoeae*; OTC, over-the-counter; POCT, point-of-care test; Tv, *Trichomonas vaginalis*; VD , vaginal discharge

Figure 2 illustrates the participant timeline.

### Data management and confidentiality

Data are collected on paper for the molecular tests (*Ct*, *Ng,* and *Tv)*, microbiological tests, Nugent scores, wet mount results, health practitioner documentation, and follow-up interviews. Researchers enter these data into a CRF using an Excel spreadsheet on the project computer. Paper versions are retained for five years. To ensure data quality, principal investigators (PIs) will conduct a random review of 10% of the data entries.

Participant interviews are conducted using C-ACASI on electronic tablets [40]. All user data is password-protected and encrypted before being transmitted to a secure web service. The encrypted data is then downloaded into a CRF on a secure project computer.

At inclusion, each participant is assigned a unique participant identification (PID) number. The connection key, linking the PID to the participant’s name and telephone number, is stored in a locked cupboard, with a backup copy kept on an encrypted USB stick. All data will be anonymized after five years.

### Statistical analysis

Descriptive analysis will be used for demographic characteristics, reproductive history, questions related to VD, clinical findings, working diagnosis by health practitioners, microbiological results, anxiety and depression scores, prevalence of DV, and prescription of medications (including antibiotics). At follow-up, we will describe symptom development, treatment adherence, and patient satisfaction for all participants after one and four months. Anxiety and depression scores will be assessed for the remaining participants at the four-month follow-up, after recruitment is completed, due to time constraints. The expected sample size is approximately 400 participants.

#### Primary outcome analyses

Two of the three primary endpoints (a) the proportion of participants overtreated with antibiotics and (b) the proportion of participants being prescribed AMR-driving antibiotics will be compared between groups (Arm 1 *vs* Arm 2 + 3). The third primary endpoint, (c) the proportion adhering to treatment recommendations at 1 month follow-up, will be compared between groups Arms 2 vs 3. The risk difference and corresponding 95% confidence intervals, as well as p-values calculated from chi-squared tests, will be reported. *P*-values below 0.017 will be regarded as statistically significant due to there being three primary outcomes.

#### Secondary outcome analyses

The binary secondary outcomes will be analysed using the same strategy as for the primary outcomes. An overall comparison of symptoms of vaginal discharge will be performed by the chi square test using three ordinal categories (better, unchanged, or worse). The secondary outcome patient satisfaction is measured on a Likert scale, and an appropriate statistical strategy will be considered after a blinded review of the distribution of scores.

#### Subgroup analysis

All primary and secondary outcomes will be analyzed based on two subgroup classifications: site (referral hospital and ORCs) and *Tv* test (before and after the introduction of an additional *Tv* test). Additionally, for the secondary outcome comparing the proportion of women acquiring OTC antibiotics after POCT-guided treatment (Arm 2 vs 3), this comparison will be analyzed in the subgroup of participants who screened positive for anxiety and depression and for those screening positive for DV.

The primary and secondary analyses are planned with an intention-to-treat approach, analyzing participants according to their allocated group regardless of treatment received or protocol deviations. A per protocol analysis will be considered depending on the proportion of non-adherence to the study protocol. We will describe the number and characteristics of non-eligible, non-included, and lost-to-follow-up participants. We will also describe the number and characteristics of non-eligible, non-included, and lost-to-follow-up participants.

### Protocol non-adherence and missing data

Protocol non-adherence is defined as participants in the intervention arms (arms 2 and 3) receiving antibiotics to treat *Ct*, *Ng*, *Tv,* or BV despite negative POCT results. These cases will be described in groups according to the three antibiotic treatment categories used: (a) treatment for *Ct*, (b) treatment for *Ng,* and (c) treatment for *Tv* and BV.

The proportion of missing data will be reported, and efforts will be made to determine the reason for missingness. The primary analyses will be complete case analyses. Two of the primary endpoints are registered immediately after the intervention and will not be subject to any substantial missingness. The third primary outcome is recorded at 4 weeks post-intervention and may have a greater risk of missingness. Multiple imputation methods will be considered if this missingness exceeds 20%.

### Storage of biological specimens for molecular analysis/future use

One labeled urine and one vaginal sample will be stored in phosphate-buffered saline at minus 80 degrees for four years in a research biobank associated with DH. After this period, the samples will be destroyed.

### Monitoring

#### Coordinating center and trial steering committee

The coordinating center is the Institute of Clinical and Molecular Medicine at the Norwegian University of Science and Technology. The trial steering committee are the principal investigators: RLH (gynecologist, Norway), SuS (gynecologist, Nepal), JI (global health researcher, Norway), JEA (microbiologist), PR (qualitative research expert, Nepal), and RK (psychiatrist, Nepal), responsible for mental health-related components.

#### Adverse event reporting and harms

Minimal risk to the participants is anticipated. Some participants may feel reluctant or anxious about self-collecting samples; therefore, RAs are trained to provide instructions using a laminated guide card. Enquiring about sensitive topics like anxiety, depression, and DV may cause emotional distress. To address this, all participants are offered contact information for available support services. Participants who express suicidal ideation are referred to the psychiatry department at DH.

All adverse events are recorded and reviewed during weekly audit meetings, and an appropriate plan of action will be developed.

#### Auditing trial conduct

Trial conduct is reviewed during weekly audit meetings. However, an informal audit by an independent researcher halfway through the trial completion was conducted which was not planned initially.

### Ethical considerations and post-trial care

Prior to the start of participant inclusion, ethical approvals were obtained from the Nepal Health Research Council (NHRC), the Institutional Review Committee of Kathmandu University School of Medical Sciences (IRC-KUSMS), and the Regional Committees for Medical and Health Research Ethics (REK). Any significant amendments to the protocol, such as the addition of an extra test for *Tv* after enrollment of the first half of the participants, are implemented only after obtaining approval from the same ethical committees.

In the case of positive *Ng* results, a new cervical swab will be collected by a health practitioner for culture and AMR testing. Treatment will be adjusted accordingly based on the results.

### Access to full protocol, participant-level data, and statistical code

In line with institutional policy and funding body requirements, all research data, including metadata, will be made accessible through a national data repository (SIKT) once the data has been anonymized. Depending on academic journal requirements, the study protocol and datasets may also be made available at an earlier stage when publishing.

### Dissemination plan

We plan to publish six articles in peer-reviewed journals as per the sequence of authorship. We plan to disseminate the study results in the year 2027. Research findings will be presented at national and international conferences.

## Discussion

A syndromic management approach has long been the standard of care for women presenting with bothersome VD in LLMICs. Compared to etiological-based diagnosis, syndromic management offers advantages in affordability and applicability during the initial visit to a health facility, thereby allowing broader population coverage and helping to limit the spread of infections [15, 42]. However, its major drawback is the overtreatment with antibiotics, particularly due to poor accuracy in detecting cervical infections caused by *Ct* and *Ng*. Given the global concern about AMR, optimizing the use of antibiotics has become imperative [14].

Over the past decade, several POCTs have been developed for STIs, enabling rapid, etiological diagnosis and potentially improving STI care and prevention. Despite a WHO “call for action,” the implementation of POCTs for STI management in LLMICs has not been widely realized [14, 16].

To examine whether implementing POCTs for STIs, striking a balance between accuracy and affordability, can reduce antibiotic use, this pragmatic RCT compares a combination of POCTs with standard syndromic management in the treatment of abnormal VD. However, implementing POCTs in LLMICs faces numerous barriers beyond cost. One important barrier may be limited knowledge among women and health practitioners about VD and appropriate antibiotic use, which could hinder the acceptability and uptake of POCT-based care.

Additionally, previous studies have shown that women experiencing common mental health disorders or DV may present with somatic symptoms such as VD [3, 32–34]. Therefore, this study also explores the impact of providing education and addressing psychosocial issues on over-the-counter antibiotic purchases, symptom progression, and patient satisfaction. Implementation studies such as POCT-BRA are important for addressing these challenges and can help bridge the gap in adapting POCTs to routine healthcare systems. This study evaluates the effectiveness of combining POCTs for STIs in relation to a globally significant and real-world outcome: antibiotic overuse.

A limitation of the trial is that the POCT used for *Ct* and *N*g is currently too expensive to be widely implemented in LLMICS. This POCT serves as a proxy for similar, highly accurate tests that may become more affordable over time, particularly if demand and market competition increase. Furthermore, the trial does not include fungal infections, as they generally do not lead to severe complications. However, fungal infections may account for a proportion of cases of bothersome VD in women without any STI.

## Trial status

Recruitment began on April 22, 2024. Inclusion of 1500 participants was completed on September 25, 2025. Follow-up of the participants is scheduled to be completed by December 31, 2025.

## Supplementary Information

Additional file 1: Contains written informed consent.

Additional file 2: Table 1 in Additional file 2 is the completed SPIRIT checklist, along with the corresponding page and line numbers where each of the items listed can be found in the manuscript [43].

## Data Availability

The datasets generated during the current study will be available at the SIKT data repository (use link, look for SIKT Data repository), once data is anonymous (e.g., the unique identifier is deleted). Summaries of dataset used for analysis are made available as soon as relevant papers are published.

## Abbreviations

AMR: Antimicrobial resistance
BV: Bacterial vaginosis
C-ACASI: Color-coded Audio Computer-Assisted Self-Interview
CI: Confidence interval
CRF: Case report form
*Ct*: *Chlamydia trachomatis*
DH: Dhulikhel Hospital
DV: Domestic violence
IRC-KUSMS: Institutional Review Committee - Kathmandu University School of Medical Sciences
LLMICs: Low- and-lower-middle-income countries
NAATs: Nucleic acid amplification tests
*Ng*: *Neisseria gonorrhoea*
NHRC: Nepal Health Research Council
OPDs: Outpatient departments
ORCs: Outreach centers
OTC: Over-the-counter
PID: Participant identification
POCTs: Point-of-care tests
RA: Research assistant
REK: Regional Committees for Medical and Health Research Ethics
STIs: Sexually transmitted infections
*Tv*: *Trichomonas vaginalis*
VD: Vaginal discharge
WHO: World Health Organization
