# Supplementary material for: Implementing point-of-care tests to optimize antibiotic use for vaginal discharge: a study protocol for a randomized controlled trial in Nepal
Source: Trials. 2025 Dec 12;27:41. doi: 10.1186/s13063-025-09333-4 (PMC12817586; doi:10.1186/s13063-025-09333-4)
Supplement: Supplementary file 1 — Additional file 1: Contains written informed consent. [file 13063_2025_9333_MOESM1_ESM.pdf]

Research title : Implementing point-of-care tests and identifying barriers to reduce antibiotics for vaginal discharge: Randomied controlled trial in Nepal, POCT-BRA

Participant's (Patients) Information Sheet for WORK-PACKAGE (WP) 2+4

PURPOSE OF THE PROJECT AND WHY YOU ARE BEING ASKED TO PARTICIPATE

My name is (name of research assistant or PhD candidate) and I am a research assistant/ PhD candidate from Kathmandu University School of Medical Sciences (KUSMS). I am inviting you to participate in this study. The main purpose of the study is to develop a better strategy for treating vaginal discharge among women in Nepal. The study participants are women with vaginal discharge, visiting outpatient departments of gynaecology departments or outreach centers. As I am explaining the study, if there are words or information you do not understand, please ask me to stop and I will take time to explain

WHAT DOES THE PROJECT ENTAIL?

If you consent to participate in the study, a computer will randomly assign you to one of three groups, one of which will receive standard treatment, while the other two will receive treatment based on test results performed during their visit. One of the groups may in addition receive specific information and counselling. All participants will receive appropriate treatment if an infection is found, regardless of their assigned group.

You will be given a container to pass urine in, and swabs to take from the vagina by yourself in the toilet. Participants will be asked to provide personal information, including their mental health status and exposure to domestic violence, through a 15-minute tablet computer survey.

I will be present during the tablet survey and the doctor will see you after you answer the questions on the tablet computer. The doctor will examine you and provide treatment and offer counselling or resources based on the assigned study group and test results. We will only collect the information from the doctor about findings related to your vaginal discharge. You may need to wait for test results for up to 90 minutes and may receive a refreshment during that time.

All the information collected from you will be used in this research project but will not include your name or telephone number.

POSSIBLE ADVANTAGES AND DISADVANTAGES

An advantage of participating in the study is that you will be contributing to improving diagnosis and treatment of the most common women's health problem - vaginal discharge. All participants will be tested for infections in the vagina and may be contacted to verify that they received the correct treatment. In addition, we will follow you up after a period. If you do not wish to participate in the study, you will receive standard treatment, which is not based on test results.

Disadvantages of participating in the study include some possible discomfort during self-collection of vaginal samples, although the swabs will not cause any local physical injury. The total process may delay your consultation time by up to 90 minutes. Further, some of the questions you will be asked are personal and may feel sensitive. You have the option to skip any question. If participating causes discomfort, you can talk to me or another healthcare worker for support. Contact information will be provided for future support.

VOLUNTARY PARTICIPATION AND RIGHT TO WITHDRAW CONSENT

Participation in the project is voluntary. If you would like to participate, please sign the consent form at the end of this document. You can withdraw your consent at any time without giving a reason, and this will have no negative consequences for you or your treatment, and your health data and biological material will not be used in any further research. You can request access to the data held on you, and this will be provided within 30 days. You can also apply for your data in the project to be corrected or deleted and for your biological material to be destroyed. The right to have your data and material destroyed, deleted, or returned does not apply if the material or data are anonymised or have been included in analysis or already been published.

If you want to withdraw at a later stage or have questions about the project, you can contact the project manager (see the contact details at the end of this document).

WHAT HAPPENS TO THE DATA HELD ON YOU?

The planned end date of the project is 31.12.2034 and all personal data will be anonymised at the end of the project. You can lodge a complaint about the processing of your data to the Norwegian Data Protection Authority and the institution's Data Protection Officer.

Your name will not be recorded except in the consent forms. All data will be processed without names and personal identification numbers or other directly identifiable information. A code links you to your data. Only Dr. Sunila, Dr. Santripti Shrestha and research assistants will have access to the code list.

Research results will be published in local and international literature. It will not be possible to identify you personally in these publications.

SHARING DATA AND TRANSFERRAL ABROAD

As part of the project, your de-identified data will be referred to Norway for analysis. The Norwegian University for Science and Technology is responsible for ensuring that data are transferred in accordance with Norwegian law and the EU General Data Protection Regulation (GDPR). The code linking you to your personally identifiable information will not be disclosed. By consenting to participate in this study, you agree to the storage of your anonymous study data at the Norwegian center for research data (NSD) and at Division of Research and Development, KUSMS for an indeterminate time. You also agree that your data may be used for further research purposes as approved by the Norwegian Ethical Committee and NHRC/KUSMS-IRC

WHAT HAPPENS TO SAMPLES TAKEN FROM YOU?

One vaginal sample and one urine sample taken from you will be stored in a research biobank associated with the project for a period of four years and we will perform tests for inflammation for scientific purposes. The results of these tests will not affect your health or management. The biobank will be closed upon completion of the project and the samples destroyed. It is the local project manager, Dr. Sunila Shakya who is responsible for this.

FOLLOW-UP PROJECT

We will follow up your appointment day with contact by telephone 4 weeks and 4 months later.

APPROVALS

The Regional Committee for Medical and Health Research Ethics has considered the research ethics in the project and given its approval.

The project has also been approved as ethically sound by the Nepal Health Research Council 406-2023 and the KUSMS Institutional Review Committees.

The Norwegian University for Science and Technology and the project manager Dr. Risa Lonnee-Hoffmann are responsible for privacy and data protection in this project.

CONTACT DETAILS

If you have questions about the project or want to withdraw your participation, you can contact Dr. Sunila Shakya by email: Sunilashakya@kusms.edu.np, or telephone 00977 9803972599.

If you have questions about data protection in the project, you can contact the Data Protection Officer at the institution: thomas.helgesen@ntnu.no or by telephone: +47 93079038

Do you agree to participate in the study?

Yes

No

Funded by The Research Council of Norway

NTNU Norwegian University of Science and Technology

KATHMANDU UNIVERSITY SCHOOL OF MEDICAL SCIENCES

DHULIKHEL HOSPITAL KATHMANDU UNIVERSITY HOSPITAL

Research title : Implementing point-of-care tests and identifying barriers to reduce antibiotics for vaginal discharge:Randomised controlled trial in Nepal, POCT-BRA

Participant's (Patient's) Consent Form for WORK-PACKAGE (WP) 2+4

I HAVE BEEN INFORMED ABOUT THE PROJECT AS MENTIONED IN THE INFORMATION SHEET GIVEN TO ME BY THE RESEARCHER.I HAVE READ OR LISSTENED ALL ABOUT IT. I GOT THE CHANCE TO CLEAR ALL MY QUERIES REGARDING THIS. I AGREE TO PARTICIPATE IN THE PROJECT AND CONSENT TO MY PERSONAL INFORMATION AND BIOLOGICAL MATERIAL BEING USED AS DESCRIBED.

Participant's signature

Participant's name in block capital letters

Participant's Index fingerprint (left finger)

I confirm that the information about the project was well provided to the participant as outlined in the document.

……………………………………………………………………………………..

Witness's signature and Name

I confirm that I have provided information about the project.

Place and date

Signature

Role in the project
